# New Estimates of Incidence of Encephalitis in England

**DOI:** 10.3201/eid1909.130064

**Published:** 2013-09

**Authors:** Julia Granerod, Simon Cousens, Nicholas W.S. Davies, Natasha S. Crowcroft, Sara L. Thomas

**Affiliations:** Public Health England, London, UK (J. Granerod);; London School of Hygiene and Tropical Medicine, London (S. Cousens, S.L. Thomas);; Chelsea and Westminster Hospital, London (N.W.S. Davies);; Public Health Ontario, Toronto, Ontario, Canada (N.S. Crowcroft)

**Keywords:** encephalitis, incidence studies, viral infections, autoimmune diseases, acute disseminated encephalomyelitis, neurological, viruses

## Abstract

Encephalitis causes high rates of illness and death, yet its epidemiology remains poorly understood. To improve incidence estimates in England and inform priority setting and treatment and prevention strategies, we used hospitalization data to estimate incidence of infectious and noninfectious encephalitis during 2005–2009. Hospitalization data were linked to a dataset of extensively investigated cases of encephalitis from a prospective study, and capture–recapture models were applied. Incidence was estimated from unlinked hospitalization data as 4.32 cases/100,000 population/year. Capture–recapture models gave a best estimate of encephalitis incidence of 5.23 cases/100,000/year, although the models’ indicated incidence could be as high as 8.66 cases/100,000/year. This analysis indicates that the incidence of encephalitis in England is considerably higher than previously estimated. Therefore, encephalitis should be a greater priority for clinicians, researchers, and public health officials.

Encephalitis is associated with severe illness, appreciable mortality rates, and high health care costs (*1*), but its epidemiology remains poorly understood (*2*). The sole previous incidence estimate for encephalitis in England of 1.5 cases per 100,000 population per year was for viral encephalitis only and was based on hospitalization data from 1989–1998 (*3*). Incidence should be understood; as an increasing number of viruses have been found to cause encephalitis in humans, more cases might be found among the high proportion of cases of unknown etiology (*2*,*4*–*6*). Climate change and increasing international travel raise the possibility of wider geographic spread of microbes, which may have important public health implications. Clarifying incidence is also important clinically with the increasing recognition of novel immune-mediated forms of encephalitis, especially because treatment is available if started early (*7*–*9*).

Although encephalitis is a statutorily notifiable disease in England, it is grossly underreported, making notification data unsuitable for incidence estimation (*3*). Almost all encephalitis case-patients require hospitalization; thus, routinely collected hospitalization data provide a possible source of data from which to estimate incidence. However, diagnosis of encephalitis is complicated by the lack of a standard case definition or pathognomonic symptoms and signs. Many patients with suspected encephalitis ultimately are found to have conditions with neurologic signs that mimic encephalitis. We recently reported on the Public Health England (PHE) study, the largest population-based prospective cohort of encephalitis patients to date in England (*10*). Data from this study, which included exhaustive multistage diagnostic investigations of cases, provided a unique opportunity to complement routinely collected hospitalization data to enable detailed analyses of encephalitis incidence in England. We linked the 2 data sources and performed capture–recapture analyses to estimate the number of encephalitis cases in England attributable to infectious and noninfectious causes.

## Methods

### Data Sources

#### PHE Study

Patients with encephalitis were recruited over a 2-year period from 24 hospitals in 3 regions of England. Suspected cases were actively identified and investigated by using extensive systematic laboratory testing, a specific case definition, and classification of cases by a multidisciplinary expert panel. Details are described elsewhere (*10*).

#### Hospital Episode Statistics 

These routinely collected data include all admissions to National Health Service hospitals in England, recorded by financial year (April–March). Each record represents an episode, a continuous period of hospital care under 1 consultant; patients may have multiple episodes within 1 hospitalization. For example, 2 separate records and hence episodes are generated in Hospital Episode Statistics (HES) if a patient is initially admitted to Accident and Emergency and subsequently transferred to a neurology ward. Each episode has up to 20 diagnoses, recorded by using codes from the International Classification of Diseases, 10th Revision (http://apps.who.int/classifications/icd10/browse/2010/en). The primary diagnosis usually reflects the main reason for hospital admission. However, incident encephalitis cases can also be recorded in secondary diagnostic fields if the primary diagnosis was a symptom or sign, if encephalitis was initially misdiagnosed, or if encephalitis developed during hospital admission.

All episodes with an admission date from April 1, 2005 through February 28, 2009 containing an encephalitis code (Technical Appendix Table 1) in any diagnostic field were obtained from HES. Data were obtained in May 2009; data from 2008–2009 were provisional and did not include cases for March 2009.

Data with an encephalitis code in any diagnostic field (the main analysis), as well as data with an encephalitis code in the primary diagnostic field only, were examined (see Table 1 for definition of numerators). Mid-2007 resident population estimates for England from the Office for National Statistics were used as denominators. Bootstrap resampling of hospital trusts and 1,000 repetitions were used to obtain 95% CIs.

**Table 1:** Definition of numerator for encephalitis incidence estimates, England

First-ever episode*	Subsequent episodes
Encephalitis code in any diagnostic field (1–20)	Subsequent encephalitis code/s represented a different etiology to that recorded for the first-ever episode; for identical codes, both were in the primary diagnostic field and the end date of the first episode and start date of the subsequent spell were >6 mo apart
Encephalitis code in primary diagnostic field only	Subsequent encephalitis codes were also in the primary diagnostic field and represented a different etiology to that recorded for the first-ever episode; for identical codes, both were in the primary diagnostic field and the end date of the first spell and start date of the subsequent spell were >6 mo apart

*Refers to the first-ever episode within this dataset.

Rates of HES encephalitis admissions were calculated by year, sex, age, and region by using data with an encephalitis code in any diagnostic field. We performed multivariable negative binomial regression analyses to investigate how the rate of incident encephalitis admissions varied with each variable.

### Capture–Recapture Analyses

For these analyses, PHE and HES data were restricted to patients admitted from November 1, 2006, through October 31, 2007, when all 24 hospitals in the PHE study were recruiting simultaneously, and to the 19 HES hospital trusts that included the 24 PHE study hospitals (hospital data are coded to trust level in HES). Cases were linked by date of birth, date of admission, sex, and postcode. Fuzzy (approximate) matching was also performed to allow for possible coding errors in the matching variables.

For 2 PHE study hospitals, the corresponding HES hospital trust included another hospital that had not participated in the PHE study and could have admitted encephalitis patients (e.g., had an accident and emergency department). Data from these 2 PHE hospitals and HES trusts were excluded in sensitivity analyses to assess their effect on capture–recapture estimates.

We determined the number of cases that matched between the 2 datasets and the numbers included only in the PHE study and only in the HES data. Fisher exact and χ^2^ tests were used to compare characteristics (including age, sex, ethnic group, and etiology) of PHE study cases that matched and did not match to HES cases and characteristics of matched and unmatched HES admissions. Other comparisons between matched and unmatched HES cases included method of admission (e.g., elective or emergency), consultant specialty, and discharge destination. The 2-sample Wilcoxon rank sum test was used to compare length of hospital stay between matched and unmatched HES admissions.

Two-sample capture–recapture analyses were performed. The Lincoln-Peterson formula was used to estimate the number of encephalitis cases that occurred from November 1, 2006, through October 31, 2007, for the 24 included sites, which comprised the number listed in either source plus the estimated number of unlisted cases (*11*). The completeness of ascertainment (%) for each data source was estimated by using this total capture–recapture estimate. CIs were calculated from the standard errors of coefficients obtained from fitting a log linear model. To assess potential violation of the capture–recapture assumption that every patient has the same probability of being ascertained by each source, data were stratified by 2 characteristics that could affect the likelihood of being ascertained: age (<18 years and >18 years) and region (South West, London, and North West). Capture–recapture analyses were rerun for each stratum, and the results were combined and compared with those from unstratified analyses.

Another key assumption of capture–recapture analyses is the absence of false-positive cases, which could have arisen in the HES data because of misdiagnosis of nonencephalitis cases or coding errors. Sensitivity analyses were thus conducted by using a range (30%–80%) of positive predictive values (PPVs) for an HES encephalitis code to reduce the number of HES-only cases included in capture–recapture models. Within this range of PPVs, we focused on a best estimate of 54%, based on the proportion of patients with suspected cases of encephalitis who were initially screened in the PHE study and ultimately included in the study (203/379), assuming that all screened PHE cases would have been coded as encephalitis in HES (*10*).

HES admissions from November 1, 2006, through October 31, 2007, for the trusts that corresponded to PHE study hospitals represented 19% of all encephalitis admissions in England. We assumed the accuracy of coding in these trusts is generalizable to all England admissions; therefore, we multiplied the capture–recapture estimate of the number of cases in these trusts by 5.3 to obtain ascertainment-adjusted national incidence estimates.

## Results

### HES Incidence

Annual incidence estimates are displayed in the Figure. On the basis of HES data with an encephalitis code in any diagnostic field, the overall mean incidence was 4.32 cases/100,000 population/year (95% CI 3.74–4.96/100,000/year). Two percent of patients (n = 216) had >1 encephalitis admission during the study period; incidence did not change (4.20 cases/100,000/year) when subsequent admissions of these patients were excluded from the analysis. By using data restricted to the primary diagnostic field, the overall mean incidence was 2.75 cases/100,000/year (95% CI 2.39 cases–3.10/100,000/year).

**Figure:**
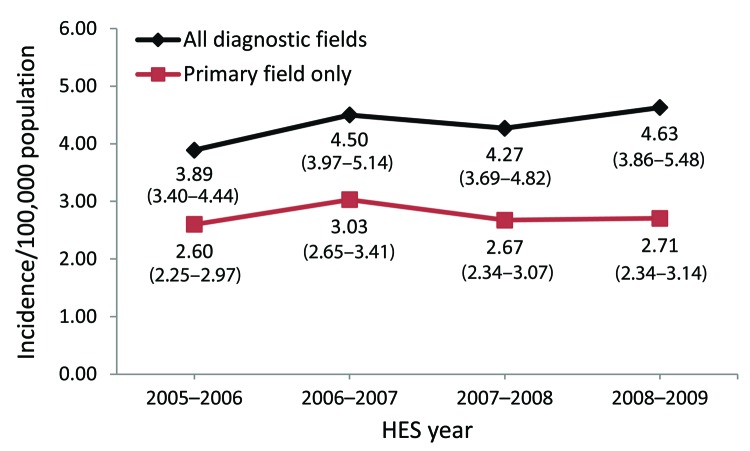
Rate of incident encephalitis admissions by year per 100,000 population, England, April 2005–February 2009. Values indicated are rate (95% CI). Overall rate for all diagnostic fields: 4.32 (3.74–4.96); for primary field only: 2.75 (2.39–3.10).

Results of multivariable analyses showed that, compared with 2005–2006, incidence in all subsequent years was slightly higher but with little evidence of a trend (p = 0.19). The incidence rate was highest among patients <1 and >65 years of age. The rate for female patients (adjusted for year, age, and region) was 8% less than the adjusted rate for male patients (Technical Appendix Table 2)

### Capture–recapture Analyses

For the defined 1-year period, the overall number of incident encephalitis admissions recorded in the relevant HES trusts was almost 4 times the number of cases in the PHE study (439 admissions vs. 113 cases) by using HES data with an encephalitis code in any diagnostic field and was 2.5 times the number (287 admissions vs. 113 cases) when HES data were restricted to the primary diagnostic field.

Fifty-nine (52%) of 113 PHE study cases matched to HES cases, and 1 case was linked by using fuzzy matching. Fifty-four cases were registered only by the PHE study and 380 were registered only by HES. The characteristics of the 59 matched HES cases and the 380 unmatched HES cases are presented in Table 2. A high proportion (91%) of encephalitis cases of unknown etiology indicated in the HES data were unmatched. Of the 125 case-patients admitted electively and 38 case-patients treated by neurosurgery in the HES data, 95% and 100%, respectively, did not match the PHE data; unmatched HES patients with admissions also had a shorter length of hospital stay (11 vs. 31 days; p<0.001; Table 2). The results were similar when HES data restricted to the primary diagnostic field were used (data not shown).

**Table 2:** Characteristics of HES-only admissions compared with those that matched to PHE study cases and characteristics of cases included in the PHE study only compared with those of PHE study cases that matched to HES, England, November 1, 2006–October 31, 2007*

Characteristics	HES				PHE study		
	HES only, n = 380	Matched,† n = 59	p value		PHE only, n = 54	Matched,‡ n = 59	p value§
Age group†							
<1	14 (77.8)	4 (22.2)	0.11§		5 (55.6)	4 (44.4)	0.22
1–4	23 (71.9)	9 (28.1)			5 (38.5)	8 (61.5)	
5–19	51 (86.4)	8 (13.6)			6 (40)	9 (60)	
20–44	113 (85.6)	19 (14.4)			15 (44.1)	19 (55.9)	
45–64	115 (88.5)	15 (11.5)			9 (39.1)	14 (60.9)	
>65	52 (92.9)	4 (7.1)			14 (73.7)	5 (26.3)	
Sex							
M	192 (86.9)	29 (13.1)	0.89§		34 (54)	29 (46)	0.18
F	188 (86.2)	30 (13.8)			20 (40)	30 (60)	
Ethnicity**							
White	248 (86.7)	38 (13.3)	0.2§		40 (48.8)	42 (51.2)	0.66
Mixed	2 (100)	0			2 (50)	2 (50)	
Asian or Asian British	23 (82.1)	5 (17.9)			1 (16.7)	5 (83.3)	
Black or black British	27 (87.1)	4 (12.9)			8 (50)	8 (50)	
Chinese or other	12 (66.7)	6 (33.3)			2 (50)	2 (50)	
Etiology							
Herpes simplex virus	13 (46.4)	15 (53.6)	<0.001§		8 (33.3)	16 (66.7)	0.01
Varicella zoster virus	25 (89.3)	3 (10.7)			2 (50)	2 (50)	
Other specified viral	37 (88.1)	5 (11.9)			3 (50)	3 (50)	
Bacterial	10 (71.4)	4 (28.6)			12 (80)	3 (20)	
Parasitic	7 (100)	0			2 (100)	0	
Amoebic	0	0			0	0	
Fungal	0	0			0	0	
Immune-mediated	37 (82.2)	8 (17.8)			5 (21.7)	18 (78.3)	
Unknown	218 (91.2)	21 (8.8)			21 (56.8)	16 (43.2)	
Other mixed	33 (91.7)	3 (8.3)			1 (50)	1 (50)	
Admission type							
Elective	119 (95.2)	6 (4.8)	0.001§				
Emergency	218 (83.5)	43 (16.5)					
Transfer††	43 (81.1)	10 (18.9)					
Discharge destination‡‡							
Home	272 (86.3)	43 (13.7)	0.85§				
Other hospital/nursing home	37 (84.1)	7 (15.9)					
Died	40 (85.1)	7 (14.9)					
Treatment specialty							
Neurosurgery	38 (100)	0	<0.001¶				
General medicine	65 (81.2)	15 (18.8)					
Neurology	121 (90.3)	13 (9.7)					
Pediatrics	27 (73)	10 (27)					
Pediatric neurology	22 (75.9)	7 (24.1)					
Infectious diseases	11 (68.8)	5 (31.2)					
Intensive care medicine	10 (71.4)	4 (28.6)					
Other	86 (94.5)	5 (5.5)					
Median length of stay, d (range)	11 (0–737); n = 351	31 (0–414);§§ n = 57	<0.001#				

*Values are no. (%) except as indicated. HES, Hospital Episode Statistics; PHE, Public Health England. †Using data from HES. ‡Using data from the PHE study. §Fisher exact test. ¶χ^2^ test. #Mann Whitney test. **For HES only. n = 312; HES matched, n = 53; PHE only n = 53. ††Transfer from other hospital, excluding emergency. ‡‡For HES matched, n = 57. §§For HES only, n = 368.

Heterogeneity in etiology was evident between unmatched PHE cases and HES-matched PHE cases (p = 0.01; Table 2). A high proportion (80%) of bacterial encephalitis cases in the PHE data were unmatched, whereas a lower proportion of PHE cases designated as immune-mediated encephalitis (22%) or herpes simplex virus (HSV) encephalitis (33%) were unmatched. Further comparison of the etiologic classification of the 59 cases that matched in the 2 datasets showed some discrepancies (Technical Appendix Table 3); 1 HES varicella zoster virus encephalitis case was diagnosed as HSV encephalitis in the PHE study, and 2 “other mixed” HES cases were diagnosed as immune-mediated in the PHE data. The 21 HES encephalitis admissions of unspecified etiology that matched to PHE study cases included 8 cases also classified as unknown etiology in the PHE study, 8 as immune-mediated, 2 as HSV, and 3 as due to other infectious causes.

The initial capture–recapture model estimated that 348 cases of encephalitis were not registered in either data source, giving an estimated number of encephalitis cases for the relevant period in that area of 841 (Table 3) and an ascertainment-adjusted incidence rate of 8.66 cases/100,000 population/year. The completeness of the 2 sources was estimated at 52% for HES and 13% for the PHE study. The estimated number of cases for children and adults combined following the stratified analyses (n = 830) was similar to the overall figure obtained in the crude analysis (n = 841); similar results were obtained when the data were stratified by region and recombined. When HES data were restricted to an encephalitis-specific code recorded in the primary diagnostic field, the capture–recapture model estimated 265 unlisted and 612 total encephalitis cases, giving an incidence estimate of 6.3 cases/100,000/year and estimated completeness of HES and the PHE study of 47% and 18%, respectively (Table 3). Similar incidence estimates were obtained after excluding from analyses the 2 sites for which additional hospitals with potential encephalitis patients were included in the HES data only (data not shown).

**Table 3:** Two-source capture–recapture estimates of encephalitis cases in England, November 1, 2006–October 31, 2007*

Coding and strata	HES only	PHE study only	Both sources	Estimated total no. cases (95% CI)	% Completeness (95% CI)	
					HES	PHE study
Encephalitis-specific code in any of the 20 diagnostic fields						
Crude analysis	380	54	59	841 (692–991)	52 (43–61)	13 (10–17)
Stratified by age group						
Children <18 y	78	15	20	172 (121–223)	57 (41–73)	20 (12–28)
Adults >18 y	290	39	39	658 (512–805)	50 (39–61)	12 (8–15)
Total for strata	368	54	59	830 (675–986)		
Stratified by region						
London	149	23	16	403 (254–552)	41 (26–56)	10 (5–14)
North West	168	29	38	364 (286–442)	57 (45–69)	18 (13–24)
South West	63	2	5	96 (49–143)	71 (38–100)	7 (1–14)
Total for strata	380	54	59	863 (689–1,038)		
Encephalitis-specific code in primary field only						
Crude analysis	234	60	53	612 (494–731)	47 (38–56)	18 (14–23)
Stratified by age group						
Children <18 y	49	16	19	126 (87–165)	54 (38–71)	28 (17–39)
Adults >18 y	173	44	34	475 (358–593)	44 (33–55)	16 (11–21)
Total for strata	222	60	53	601 (478–725)		
Stratified by region						
London	86	23	14	265 (159–371)	38 (22–53)	14 (7–21)
North West	114	29	34	275 (211–339)	54 (42–66)	23 (16–30)
South West	34	2	5	55 (27–83)	71 (38–100)	13 (2–23)
Total for strata	234	54	53	595 (469–722)		

*HES, Hospital Episode Statistics; PHE, Public Health England.

Results of sensitivity analyses to account for potential overdiagnoses of HES-only encephalitis cases are reported in Table 4. By assuming a best estimate PPV of 54% for HES-only admissions, the capture–recapture model estimated 508 total cases, giving an incidence of 5.23 cases/100,000/year and an estimated completeness of 22% for the PHE study; this figure represents our “best estimate” of the true incidence of encephalitis in England. By assuming a higher PPV for HES-only admissions, the number of estimated total cases increased and the completeness of the PHE study decreased. When HES data were restricted to the primary diagnostic field only and a PPV of 54% was assumed, the capture–recapture model estimated 384 total cases, equivalent to an incidence of 3.96 cases/100,000/year (Table 4).

**Table 4:** Sensitivity analysis adjusting for variation in positive predictive value for HES-only admissions*

Positive predictive value, %	HES only	PHE study only	Both sources	Estimated total no. cases (95% CI)	% Completeness (95% CI)		Estimated incidence
					HES†	PHE study	
Encephalitis-specific code in any of the 20 diagnostic fields							
30	114	54	59	332 (272–391)	52 (43–61)	34 (27–41)	3.42
40	152	54	59	405 (332–477)	52 (43–61)	28 (22–34)	4.17
50	190	54	59	477 (392–562)	52 (43–61)	24 (18–29)	4.91
54	206	54	59	508 (418–598)	52 (43–61)	22 (17–27)	5.23
60	228	54	59	550 (452–648)	52 (43–61)	21 (16–25)	5.67
70	266	54	59	623 (512–734)	52 (43–61)	18 (14–22)	6.42
80	304	54	59	696 (572–819)	52 (43–61)	16 (12–20)	7.17
Encephalitis-specific code in primary field only							
30	71	60	53	265 (214–315)	47 (38–56)	43 (34–51)	2.73
40	94	60	53	314 (254–374)	47 (38–56)	36 (28–44)	3.23
50	117	60	53	363 (293–433)	47 (38–56)	31 (24–38)	3.74
54	127	60	53	384 (310–458)	47 (38–56)	29 (23–36)	3.96
60	141	60	53	414 (334–494)	47 (38–56)	27 (21–34)	4.26
70	164	60	53	463 (374–553)	47 (38–56)	24 (19–30)	4.77
80	188	60	53	514 (415–614)	47 (38–56)	22 (17–27)	5.29

*Incidence = cases/100,000 population. HES, Hospital Episode Statistics; PHE, Public Health England †Due to the Lincoln-Petersen formula, if the number of HES-only admissions varies, the total HES admissions (HES only plus matched) is proportional to the estimated total number of cases; hence, the completeness of HES (ratio) does not change with varying positive predictive value.

## Discussion

Our analyses provide estimates of the incidence of encephalitis in England attributable to infectious and noninfectious causes. We present a unique application of capture–recapture models to estimate the number of cases of encephalitis by using an original dataset of well-defined and extensively investigated cases of encephalitis.

Multiple scenarios were investigated to assess the sensitivity of the estimates to various assumptions. Depending on the scenario, estimated incidence ranged from 2.73 cases/100,000/year to 8.66 cases/10^5^/year; all estimates were higher than the 1.5 cases/100,000/year previously reported (*3*). This unique study has brought together 2 distinct datasets to help address the inevitable limitations within such data sources, particularly those encountered with complex syndromes such as encephalitis. We consider our capture–recapture estimate of 5.23/100,000/year (assuming 54% PPV for HES data) to be the best estimate of encephalitis incidence in England; this is 3.5 times higher than that previously described by Davison et al (*3*). Our incidence analyses update the Davison et al. estimates; diagnostic advances, emerging etiologic agents, and introduction of new interventions and control strategies (e.g., vaccines) are all likely to have affected incidence estimates over time. Furthermore, our data included both infectious (not just viral) and noninfectious causes of encephalitis, in line with the increased recognition of new immune-mediated encephalitis etiologies.

A higher incidence (adjusted for year, age, and region) of encephalitis was observed among male patients, which is consistent with previous studies (*12*–*16*). We also observed higher incidence of encephalitis among patients <1 and >65 years of age. Hyporesponsiveness of the immune system in early life and later immunosenescence render these groups more susceptible to infection, to reactivation of latent infection, or development of encephalitis once infected (*17*).

Multiple admissions were included in our analyses only if they represented different etiologies or occurred >6 months apart, either of which is not a common occurrence in encephalitis as supported by our data. When multiple occurrences were excluded, incidence was unchanged. Infectious episodes of encephalitis are unlikely to recur in the absence of immunosuppression; relapses in immune-mediated cases are more frequent and were documented in the PHE study.

We extended our encephalitis incidence analyses beyond the use of HES and linked HES data to PHE study data and applied capture–recapture models. The number of incident encephalitis admissions recorded in HES was considerably higher than the number of cases included in the PHE study, even after restricting HES diagnoses to the primary diagnostic field. Conversely, nearly half of the PHE cases were not captured in HES. The poor agreement between these 2 data sources could have several possible reasons.

The likely explanation for nonmatched PHE cases is that testing in the PHE study went far beyond routine clinical practice, which highlighted the extent to which encephalitis can be underdiagnosed. The higher proportion of bacterial cases in the PHE study, classified as meningoencephalitis, suggests that these cases may be coded as meningitis rather than encephalitis in HES. Also, patients with unusual signs and symptoms, such as those with N-methyl-D-aspartate receptor-antibody encephalitis, which typically causes psychiatric symptoms, might not have been classified as encephalitis case-patients in HES (*18*). Unfortunately, we could not identify the HES codes used for the unmatched PHE cases because the HES data for this study included only patients with an encephalitis code.

Unmatched HES cases likely include true encephalitis cases not reported to the PHE study team and nonencephalitis cases misdiagnosed as encephalitis. Underascertainment in the PHE study is likely, as employing >1 research nurse per region to actively identify cases was not financially feasible: some centers relied on case notification by hospital staff alone. The likelihood that HES admissions coded as encephalitis included misdiagnoses of syndromes with signs that mimic encephalitis is highlighted by the PHE study, in which only 54% of suspected encephalitis patients initially screened during the 2-year period were ultimately included following a rigorous diagnostic process (*10*). The higher proportion of cases of unknown etiology in unmatched compared with matched HES admissions and their shorter length of hospital stay supports the possibility of misdiagnosis and suggests a more likely diagnosis of a mimicker syndrome such as septic encephalopathy.

Other reasons for mismatches need consideration. The catchment areas covered by the 2 data sources differed slightly, but the results of the capture–recapture model did not change when we excluded the 2 trusts that had hospitals with potential extra encephalitis cases in the HES data. The higher number of admissions in HES could be due in part to the inclusion of patients with postencephalitic sequelae, who would not have been notified to the PHE study. This finding is supported by the higher proportion of cases admitted electively and treated under neurosurgery in unmatched HES admissions; alternatively, some of these cases could be miscoded nonencephalitis mimics, i.e., cases of nosocomial meningitis following surgery.

Three assumptions required for valid capture–recapture estimates also need consideration. First, for a given source every patient should have an equal chance of being ascertained by that source, although different sources may have different probabilities of identifying an individual case. Our analyses stratified by age or region of residence did not indicate any bias in the point estimate linked to these variables. Other variables may have influenced the probability of a patient appearing on a list, such as ethnicity, sociodemographic factors, or heterogeneity in coding between hospitals within regions. Because of the small sample sizes, we could not stratify by these variables. We did find evidence of heterogeneity in etiology, which suggested that in 1 or both datasets, patients with bacterial encephalitis had a different probability of being identified than did patients with encephalitis of other etiologies, which may have led to an overestimate of the number of encephalitis cases. Again, performing a stratified analysis is not easy, both because of small numbers and because we know misclassification and missing data about etiology were present and no pathognomonic features exist to allow different causes to be distinguished in these cases.

Second, the registers used in capture–recapture should be independent; having an encephalitis-specific diagnosis in HES should not affect being included in the PHE study or vice versa. A patient with suspected encephalitis seen by a hospital clinician was likely to be coded as such in HES and also be notified to the PHE study. Thus, these sources are likely to be positively dependent, and the capture–recapture will have underestimated the true number of cases. A less likely scenario is that the datasets were negatively dependent, for example, if encephalitis cases with bacterial etiology were simultaneously more likely to be excluded from the HES data and more likely to be included in the PHE data. This situation would have led to an overestimate in the number of cases. We did not have access to a third data source to evaluate the independence of data sources (*19*). Nevertheless, 2-source capture–recapture can indicate an upper or (as is likely here) the lower bound of estimates when the direction of dependency is known or highly suspected (*20*).

Third, no false positive cases should occur due to misdiagnoses or miscoding. As discussed above, coded HES encephalitis cases are likely to have included mimicker syndromes, which would inflate the capture–recapture estimate. We addressed this possibility by applying a range of PPVs to the HES data; even after assuming a PPV as low as 30% for HES-only admissions, the number of cases was still higher than previous estimates. A review of medical records would be necessary to determine the degree of misdiagnosis of true encephalitis cases in HES data. We could not do this because HES do not keep patients’ names, and use of HES data are subject to strict protocols to prevent identification of individual patients.

With a mean length of hospital stay of 34 days, an incidence of 5.23 cases/100,000/year (“best estimate”) equates to 90,852 bed-days of hospital occupancy. On the basis of a bed-day cost of £261 (US$394 million), the cost to the National Health Service would be >£23 million (US$35 million) per year (*21*). An incidence of 8.66 cases/100,000/year, our maximum estimate, would cost almost £40 million (US$60 million) per year. The actual cost is likely to be higher as patients often require intensive care, costly investigations, and in-patient rehabilitation. Additional costs include long-term care and loss of productivity among many working-age survivors.

In summary, the different scenarios used in this study provide strong evidence that the incidence of encephalitis is higher than that previously estimated in England. This higher incidence has clinical, research, and public health implications. A diagnosis of encephalitis should be considered for patients with compatible symptoms, especially given the increased recognition of immune-mediated encephalitides for which treatment is available and effective if instigated early. Early recognition is important to help reduce the substantial economic and societal costs of encephalitis suggested by our study. Stand-alone HES data are used extensively for public health research; our analyses highlight the extent to which HES-only data might over- or underascertain cases of complex syndromes and the advantages of linking these data to other sources to improve incidence estimates. Encephalitis incidence in this study was higher than that of other neurologic conditions, such as meningococcal meningitis and motor neuron disease, both of which have a higher profile and public focus (*22*–*24*). This study highlights the importance of accurate diagnosis and coding for complex syndromes with multiple etiologies to obtain accurate estimates of incidence and to further explore the epidemiology and outcomes of this devastating neurologic illness.

Technical Appendix Tables showing Hospital Episode Statistics (HES) codes for identifying encephalitis; independent effect of year, sex, age, and region on incident encephalitis admissions; comparison of the cause of 59 cases that matched between Public Health England and HES data.
