# Supplementary material for: New Estimates of Incidence of Encephalitis in England
Source: Emerg Infect Dis. 2013 Sep;19(9):1455–62. doi: 10.3201/eid1909.130064 (PMC3810913; doi:10.3201/eid1909.130064)
Supplement: Technical Appendix — Tables showing Hospital Episode Statistics (HES) codes for identifying encephalitis; independent effect of year, sex, age, and region on incident encephalitis admissions; comparison of the cause of 59 cases that matched between Public Health England and HES data. [file 13-0064-Techapp-s1.pdf]

Article DOI: http://dx.doi.org/10.3201/eid1909.130064

High Estimated Incidence of Encephalitis in England

Technical Appendix

Technical Appendix Table 1. Hospital Episode Statistics codes for identifying encephalitis and etiological groupings*

Infection type	Codes
Viral	
Herpes simplex virus	B004
Varicella-zoster virus	B011, B020
Enterovirus	A850
Adenovirus	A851
Measles	B050
Mumps	B262
Rabies	A820 A821 A829
Arboviruses	A830 A831 A832 A833 A834 A835 A836 A922 A838 A839 A852 A840 A841 A848 A849
Rubella	B060 AND G051
Influenza	J108/J118 AND G051
Polio	A800/A801/A802/A803/A804/A809 AND G051
Lymphocytic choriomeningitis virus	A872 AND G051
Cytomegalovirus	B258 AND G051
HIV	B220 AND G051
Progressive multifocal leukoencephalopathy	A812
Other specified viral encephalitis (encephalitis lethargica, Von Economo-Cruchet disease)	A858
Subacute sclerosing panencephalitis	A811
Bacterial	
Listeria	A321 AND G050
Meningococcal infection	A398 AND G050
Syphilis	A504/A521 AND G050
Tuberculosis	A178 AND G050
Streptococcal infection	A491 AND G050
Lyme disease	A692 AND G050
Cat-scratch disease	A281 AND G050
Actinomycosis	A428 AND G050
Bacterial meningoencephalitis and meningomyelitis, not elsewhere classified	G042
Parasitic	
Trypanosomiasis	B560 B561 B569 B574
Toxoplasma	B582
Amoebic	
Naegleriasis (primary amoebic meningoencephalitis)	B602
Angiostrongyliasis due *to Parastrongylus cantonensis* (eosinophilic meningoencephalitis)	B832
Fungal	
Cryptococcosis	B451 AND G052
Immune-mediated	
Acute disseminated encephalomyelitis	G040
Acute and subacute haemorrhagic leukoencephalitis	G361
Systemic lupus erythematosus	M321 AND G058
Paraneoplastic limbic encephalopathy	G131
Myxoedema coma	
Unknown	
Unspecified viral encephalitis	A86; G050 G051 G052 G058 IF no other code specified
Encephalitis, myelitis, and encephalomyelitis, unspecified—ventriculitis (cerebral) not otherwise specified	G049
Other (includes other encephalitis, myelitis and encephalomyelitis,postinfectious encephalitis, and encephalomyelitis not otherwise specified)	G048

Technical Appendix Table 2. Main effects negative binomial regression model assessing variation in incident encephalitis admissions by year, sex, age, and region, England, April 2005–February 2009*

Level	No. (%) incident admissions, n = 8,848	Crude incidence per 100,000 population/year	Multivariable		
			Adjusted IRR	95% CI	p value
Year					
2005–6	1,991 (22)	3.89 (3.40–4.44)	1.00	–	0.002
2006–7	2,304 (26)	4.5 (3.97–5.14)	1.15	1.07–1.24	
2007–8	2,176 (25)	4.27 (3.69–4.82)	1.09	1.02–1.18	
2008–9	2,369 (27)	4.63 (3.86–5.48)	1.07	1.00–1.15	
Missing	8 (0.1)				
Sex					
Male	4,525 (51)	4.50 (3.93–5.14)	1.00	–	0.002
Female	4,322 (49)	4.15 (3.61–4.75)	0.92	0.88–0.97	
Missing	1 (0.0001)				
Age group					
<1	300 (3)	11.63 (9.75–13.89)	3.66	3.22–4.17	<0.001
1–4	581 (7)	6.06 (5.14–7.13)	1.89	1.71–2.09	
5–19	1,077 (13)	2.86 (2.43–3.39)	0.91	0.84–0.99	
20–44	2,326 (27)	3.23 (2.78–3.80)	1.00	–	
45–64	2,259 (26)	4.46 (3.86–5.08)	1.41	1.31–1.51	
≥65	1,973 (23)	6.06 (5.28–6.75)	1.94	1.80–2.08	
Missing	332 (4)				
Region					
North West	1,222 (14)	4.33 (2.64–6.49)	1.00	–	<0.001
South West	879 (10)	4.22 (2.66–6.10)	0.93	0.84–1.03	
London	1,692 (19)	5.51 (3.93–7.61)	1.22	1.11–1.34	
North East	413 (4)	3.93 (1.97–6.32)	0.92	0.82–1.05	
South East	1,272 (15)	3.76 (2.61–5.29)	0.87	0.79–0.95	
West Midlands	822 (9)	3.78 (2.61–5.18)	0.87	0.78–0.97	
East Midlands	710 (8)	3.99 (2.20–6.28)	0.90	0.81–1.01	
East of England	817 (9)	3.50 (2.30–5.22)	0.80	0.72–0.89	
Yorkshire and the Humber	873 (10)	4.18 (2.75–6.18)	0.84	0.76–0.93	
Missing	126 (1)				

*IRR, incidence rate ratio; HSV, herpes simplex virus; VZV, varicella- zoster virus.

Technical Appendix Table 3. Comparison of the aetiological classification of the 59 cases that matched between HES and the HPA study*

HPA diagnosis	HES diagnosis										
	HSV	VZV	Other viruses	Bacterial	Parasitic	Amoebic	Fungal	Immune-mediated	Unknown	Other mixed	Total
HSV	13	1	0	0	0	0	0	0	2	0	16
VZV	0	2	0	0	0	0	0	0	0	0	2
Other specified viral	0	0	2	0	0	0	0	0	1	0	3
Bacterial	0	0	0	2	0	0	0	0	1	0	3
Parasitic	0	0	0	0	0	0	0	0	0	0	0
Amoebic	0	0	0	0	0	0	0	0	0	0	0
Fungal	0	0	0	0	0	0	0	0	0	0	0
Immune-mediated	0	0	0	0	0	0	0	8	8	2	18
Unknown	2	0	3	2	0	0	0	0	8	1	16
Other mixed	0	0	0	0	0	0	0	0	1	0	1
Total	15	3	5	4	0	0	0	8	21	3	59

*HES, Hospital Episode Statistics; HPA, Health Protection Authority;HSV, herpes simplex virus; VZV, varicella-zoster virus.
